# Personal

**Published:** 1903-06

**Authors:** 


### PERSONAL.

Dr. Henry Jones Mulford, of Buffalo, announces the removal of his office and residence from 405 Delaware Avenue to 143 Allen Street. Hours: 9 to 12 a.m.; 2 p.m.; Sunday at noon. Bell telephone, Tupper 772. Practice limited to diseases of nose, throat and ear.

---

Dr. John Chalmers, of Buffalo, announces the removal of his office and residence from 154 Chenango Street to 321 West Ferry Street. Hours: 8 to 9 a.m.; 2 to 3 and 7 p.m.; Sunday, 1 to 3 p.m.

---

Dr. Max C. Breuer, of Buffalo, announces the removal of his office and residence, from No. 1 North Pearl Street, to 33 Allen Street, corner North Pearl Street. Hours: 9 to 10 a.m.; 2 to 3 p.m. Evening by appointment.

---

Dr. Franklin W. Barrows, of Buffalo, announces his removal from 45 Park Street and 464 William Street, to his new residence and office, No. 1364 Michigan Street. Telephones, Bryant, 3911; Frontier, 26431.

---

Dr. Joseph Burke, of Buffalo, has removed his office and residence from No. 388 Franklin Street to No. 888 Main Street. Hours: 1 to 3 and 7 p.m. Practice limited to diseases of the thoracic and abdominal viscera.

---

Dr. Grosvenor R. Trowbridge, of Buffalo, has removed his office from The Wellesley, Franklin Street, to 1349 Main Street. Hours: 8 to 9; 1 to 3 and 6 to 7. Telephones: Bell, Bryant, 762; Frontier, 637.

---

Dr. A. L. Gnichtel, of New York, has removed to 257 West 44th Street. Hours: Until 10; 1 to 2; 4.30 to 7. Telephone, 3203-38th St.

---

Dr. George F. Cott, of Buffalo, has removed his residence from No. 160 Woodlawn Avenue to No. 330 Depew Avenue, Central Park. His office still remains in the Mooney-Brisbane building. Practice limited to diseases of the ear, nose and throat.

---

Dr. Richard H. Satterlee, of Buffalo, has removed from The Touraine to his former residence, 187 Delaware Ave. Hours: 9-1; 3-4. Practice limited to diseases of the eye. Telephone, Tupper 509.

---

Dr. James A. Gibson, of Buffalo, has removed his residence from 533 Franklin Street to The Touraine, Delaware Avenue, corner of Johnson Park. His office remains in the Mooney-Brisbane building.

---

Professor C. A. Ewald, of Berlin, addressed the Buffalo Academy of Medicine at a special meeting held Wednesday evening, May 20, 1903. Subject: The early diagnosis of gastric cancer. Dr. A. L. Benedict entertained the guest and members at luncheon after the meeting.

---

Dr. Charles G. Stockton, of Buffalo, tendered a reception to Professor Ewald, of Berlin, and the Fellows of the Academy of Medicine, at the Saturn Club, Wednesday, May 20, 1903, from 3 to 4 p.m.

Dr. Harvey R. Gaylord, director of the Gratwick Laboratory, University of Buffalo, who has been ill for some weeks with typhoid fever, is reported to be convalescing satisfactorily.

---

Dr. Lewis S. McMurtry, of Louisville, delivered the address on surgery before the South Carolina Medical Association, at its recent meeting in Charleston. He chose for his subject, Fibroid tumors of the uterus and their complications.

---

Dr. Chauncey Pelton Smith, of Buffalo, delivered an address before the Buffalo Historical Society in its new building Sunday afternoon, May 17, 1903, the subject of which was, Some historical curiosities of medical practice. This was one of the regular Sunday afternoon series of popular lectures and attracted an interested audience.

---

Dr. Eugene Wasdin, Surgeon U. S. public health and marine hospital service, and Mrs. Wasdin, have taken apartments at The Touraine, Buffalo. Dr. Wasdin has been stationed at Buffalo during the past two years and will be remembered as one of President McKinley's physicians.

